# Supplementary material for: Longitudinal structural connectomic and rich-club analysis in adolescent mTBI reveals persistent, distributed brain alterations acutely through to one year post-injury
Source: Sci Rep. 2019 Dec 11;9:18833. doi: 10.1038/s41598-019-54950-0 (PMC6906376; doi:10.1038/s41598-019-54950-0)
Supplement: Supplementary file 1 — Supplementary Materials [file 41598_2019_54950_MOESM1_ESM.pdf]

Supplementary Materials

Longitudinal structural connectomic and rich-club analysis in adolescent mTBI reveals persistent, distributed brain alterations acutely through to one year post-injury

Ai Wern Chung, Rebekah Mannix, Henry A. Feldman, P. Ellen Grant, Kiho Im

Analysis S1 - QBall reconstruction and global network analysis

This analysis was performed using the Diffusion Toolkit[1]. Q-Ball ODF reconstruction of the diffusion data was achieved with a spherical harmonics basis method[2] and 2nd order Runge-Kutta method employed for tractography. Tractography was seeded 20 times from each white matter voxel in the brain, with a tracking step-size of 0.1mm and angular threshold of 35°. Tracts with length greater than 20mm and less than 200mm were retained. The cortical regions reached by each tract's end point was recorded. The number of streamlines connecting any two regions formed the corresponding entry in the connectome. Network nodes included 68 cortical regions, and edge weights were the number of streamlines connecting pair-wise nodes. Connectivity matrices were normalised by the total number of tracts in the same matrix[3]. The DTI-based analysis as detailed in the main manuscript was repeated on these QBall-based ODF networks. The same network measures were computed and the same statistical analysis carried out. Results are plotted in Figure S1.1 and Table S1.1, showing great similarity in trends to those from DTI networks (Figure S4), namely betweenness centrality, transitivity, global efficiency and modularity decreased with time in patients, and values were higher in Controls when compared with the Chronic time point. Similarly for both models, the opposite trend of increasing degree with time in mTBI and lower values in Controls versus the Chronic time-point is observed. While there are differences between models in which measures reached significance in our statistical analysis, cross-sectional comparisons between mTBI and Controls remained non-significant in both models and transitivity and global efficiency measures exhibited significant changes over time for mTBI subjects. Future work with larger sample sizes will determine the significance of these trends and their consistency between models.

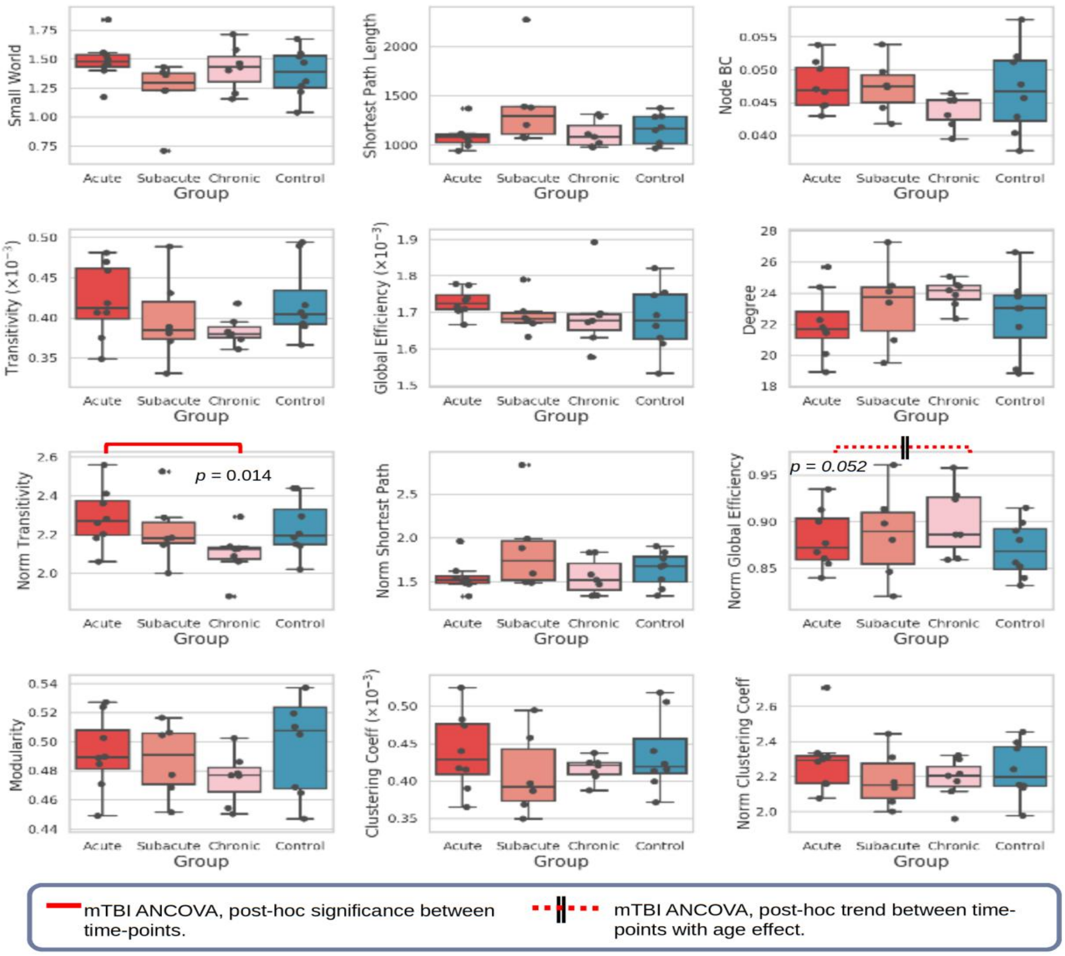

Figure S1.1 - Box plots of global network theoretical measures computed from QBall-based connectomes at each mTBI time-point and for Controls. Black circles represent individual data points. Significant post-hoc differences between time-points are shown only when the corresponding ANCOVA analysis in the mTBI cohort is significant. BC = Betweenness centrality.

		Longitudinal repeated measures ANCOVA													Cross-sectional ANCOVA											
												Post-hoc *p*-value between time points			Acute mTBI vs. Control						Chronic mTBI vs. Control					
		Age-adjusted mean						Fixed effects				Acute vs Subacute	Acute vs Chronic	Subacute vs Chronic	Age-adjusted mean		Fixed effect significance				Age-adjusted mean		Fixed effect significance			
		Acute		Subacute		Chronic		Age at MRI		Time-point					Controls		Age at MRI		Group		Controls		Age at MRI		Group	
		Mean	Std Error	Mean	Std Error	Mean	Std Error	F-statistic	*p*-value	F-statistic	*p*-value				Mean	Std Error	F-statistic	p-value	F-statistic	p-value	Mean	Std Error	F-statistic	p-value	F-statistic	p-value
	**Small world**	1.483	0.060	1.173	0.121	1.425	0.080	0.542	0.490	2.675	0.193				1.360	0.061	5.806	**0.032**	2.802	0.118	1.384	0.075	0.195	0.667	0.078	0.785
	**Shortest path**	1089.91	52.33	1046.80	280.42	1103.35	44.57	2.077	0.372	0.021	0.980				1168.74	46.86	3.145	0.100	1.802	0.202	1153.92	53.99	0.271	0.612	0.237	0.635
	**Transitivity ($x10^{-3}$)**	0.421	0.014	0.396	0.018	0.384	0.005	10.314	**0.023**	3.550	0.091				0.415	0.014	7.187	**0.019**	0.290	0.599	0.416	0.012	4.031	0.068	2.695	0.127
	**Global efficiency ($x10^{-3}$)**	1.726	0.013	1.678	0.021	1.691	0.032	0.232	0.647	4.859	*0.056*				1.679	0.026	0.343	0.568	1.863	0.195	1.685	0.034	0.475	0.504	0.003	0.961
	**Node BC**	211.66	6.318	209.46	7.322	194.250	3.986	2.096	0.212	4.629	0.069				206.624	8.689	0.344	0.568	0.131	0.723	206.003	8.030	1.302	0.276	0.825	0.381
	**Degree**	21.958	0.700	23.925	0.988	23.824	0.363	15.156	**0.026**	2.501	0.153				22.739	0.771	4.164	*0.062*	0.715	0.413	22.642	0.710	1.187	0.297	1.297	0.277
	**Modularity**	0.491	0.007	0.484	0.010	0.476	0.008	6.116	**0.048**	2.243	0.197				0.495	0.009	9.446	**0.009**	0.001	0.980	0.497	0.010	1.187	0.297	1.934	0.190
	**CC ($x10^{-3}$)**	0.440	0.015	0.412	0.019	0.417	0.005	27.987	**0.004**	1.307	0.335				0.430	0.015	8.667	**0.011**	0.449	0.514	0.432	0.013	3.723	0.078	0.438	0.520
Normalized	**Transitivity**	2.290	0.054	2.221	0.067	2.099	0.048	0.170	0.694	5.756	**0.039**		0.014		2.222	0.051	2.717	0.123	1.141	0.305	2.229	0.050	0.443	0.518	2.747	0.123
	**Shortest path**	1.553	0.086	1.440	0.329	1.547	0.063	21.200	**0.030**	0.104	0.903				1.655	0.065	2.398	0.145	1.465	0.248	1.634	0.074	0.614	0.449	0.366	0.557
	**Global efficiency**	0.878	0.014	0.892	0.015	0.901	0.011	10.936	**0.020**	6.655	**0.029**		*0.052*		0.871	0.011	0.233	0.638	0.305	0.590	0.873	0.011	2.554	0.136	2.108	0.172
	**CC**	2.296	0.066	2.185	0.061	2.188	0.049	0.222	0.654	1.253	0.358				2.216	0.057	4.443	*0.055*	1.247	0.284	2.227	0.053	0.460	0.511	0.244	0.631

Table S1.1 - Global network theory analyses computed on QBall-based connectomes. Age-adjusted means and statistical results from longitudinal (mTBI only) and cross-sectional (mTBI vs control) ANCOVA analyses. ANCOVA significance is at *p* <= 0.05, marked in **bold,** trends are *italicised*. 'Age at MRI' is at the acute time point for mTBI subjects. Normalised network measures were computed against 1000 random networks of equal density to the observed networks. BC = Betweenness Centrality; CC = Clustering Coefficient.

Analysis S2 - Rich-club computation and analysis

Analysis S2.1 Weighted networks

Rich-club (RC) analysis was performed on group-averaged, weighted connectomes[4–8]. First a binarised, group-average adjacency matrix was computed by retaining edges in at least 60% of the subjects in each group. Weights are subsequently added to the group-averaged adjacency matrix by taking the average weight of each connection across the group, generating a weighted group-averaged connectome W_{group}, where there are four group connectomes, comprising one for the Controls, and the concussion cohort at each time point.

All rich club analysis was performed using the Brain Connectivity Toolbox (https://sites.google.com/site/bctnet)[9]. W_{group} was then used to calculate the weighted RC parameter $\Phi_{group}(k)$[10], where k denotes the degree of nodes. The RC parameter $\Phi_{group}(k)$ is normalised relative to a set of comparable random networks of equal size and with similar connectivity distribution. Here, we generate 1000 random networks while preserving weight, degree and strength distributions of W_{group}[9,11]. For each of these random realisations of the graph, we calculate the weighted RC parameter $\Phi_{rand}(k)$. Finally, the normalised weighted RC parameter is calculated as

$$\Phi_{group}^{norm}\ (k) = \frac{\Phi_{norm}\ (k)}{\Phi_{rand}\ (k)}. \qquad \textbf{[Equation S1]}$$

For this metric, $\Phi_{group}^{norm}\ (k) > 1$ denotes the presence of a rich-club. In our analysis, we select

$$k_{max}^{group} : max\left(\Phi_{group}^{norm}\right), \text{ for which } \Phi_{group}^{norm}\ (k) > 1, \qquad \textbf{[Equation S2]}$$

as the degree of the RC nodes of a given group, which allows us to determine the RC members with a degree of at least k. Statistical significance of $\Phi_{group}^{norm}\ (k) > 1$ was assessed by performing a right-sided t-test for each k (p < 0.05 after Bonferroni correction, where the number of tests equals the maximum degree within each group connectome)[4].

Figure S2.1 plots the normalised RC coefficient, $\Phi_{group}^{norm}\ (k)$, with k for each group connectome computed from the DTI networks. Significance of $\Phi_{group}^{norm}\ (k)$> 1 was found for a range of degrees in each group and time-point (from k = 16 to 20), indicative of a rich-club organisation in all cohorts.

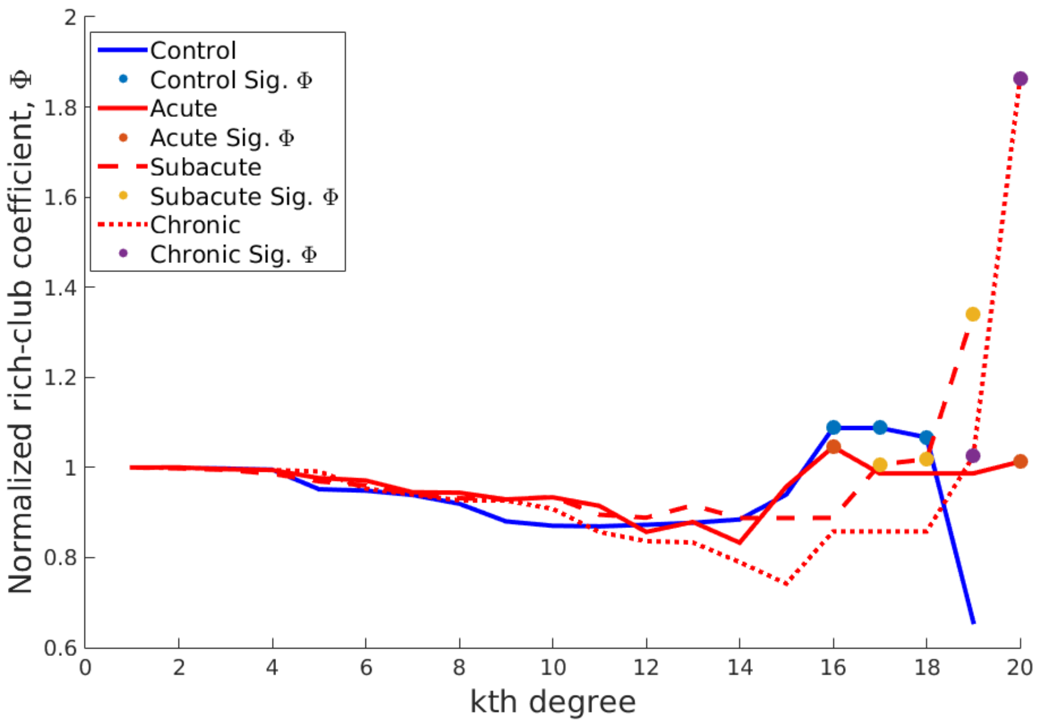

Figure S2.1 - Normalised rich-club coefficient with k degree for Controls and each mTBI time-point group, computed from DTI networks. Significance of $\Phi_{group}^{norm}(k) > 1$ are denoted by circles.

The k_{max}^{group} to define the rich-club nodes in each group were 16, 16, 19 and 20 for Controls, Acute, Subacute and Chronic, respectively. Rich-club nodes for each group are shown in Table S2.1.

Control	Acute	Subacute	Chronic
rh-insula	rh-insula	rh-precuneus	rh-superiorfrontal
rh-precuneus	rh-precuneus	rh-superiorfrontal	rh-superiorparietal
rh-superiorfrontal	rh-superiorfrontal	rh-superiorparietal	lh-superiorfrontal
rh-superiorparietal	rh-superiorparietal	lh-superiorfrontal	lh-superiorparietal
lh-insula	lh-insula	lh-superiorparietal	
lh-precuneus	lh-posteriorcingulate		
lh-superiorfrontal	lh-superiorfrontal		
lh-superiorparietal	lh-superiorparietal		

Table S2.1 - Rich-club nodes for each group computed from DTI networks

RC nodes were similarly identified from the QBall networks (from Analysis S1), with group-averaged adjacency matrices computed by retaining edges in at least 70% of the subjects in each group. A range of degrees where $\Phi_{group}^{norm}(k) > 1$ was significant were identified in each group and time-point (from k = 23 to 34), indicative of a rich-club organisation in all cohorts. The k_{max}^{group} to define the rich-club nodes in each group were 25, 27, 27 and 29 for Controls, Acute, Subacute and Chronic, respectively. Rich-club nodes for each group are shown in Table S2.2.

Control	Acute	Subacute	Chronic
rh-precuneus	rh-insula	rh-precuneus	rh-precuneus
rh-rostralmiddlefrontal	rh-postcentral	rh-superiorfrontal	rh-rostralmiddlefrontal
rh-superiorfrontal	rh-precuneus	rh-superiorparietal	rh-superiorfrontal
rh-superiorparietal	rh-superiorfrontal	lh-precuneus	rh-superiorparietal
lh-precuneus	rh-superiorparietal	lh-superiorfrontal	lh-precuneus
lh-superiorfrontal	lh-precuneus	lh-superiorparietal	lh-superiorfrontal
lh-superiorparietal	lh-superiorfrontal		lh-superiorparietal
	lh-superiorparietal		

Table S2.2 - Rich-club nodes for each group computed from QBall networks

Analysis S2.2 Binary networks

RC analysis was performed on group-based, binary connectomes. Each subject's network was binarised (1 if there was a weighted edge, 0 otherwise) and a group-average adjacency matrix was computed by retaining edges in at least 60% of the subjects in each group. $\Phi_{group}(k)$ was calculated for each binary, group network[12] and normalised against 1000 equivalent random networks of equal size and degree distribution[13]. The kth degree threshold for RC nodes was defined as in Equation S2. Rich club organisation, where $\Phi_{group}^{norm}(k) > 1$ is significant, was found for each group and time point for a range of degrees from k=4-17. The k_{max}^{group} to define the RC nodes in each group were 11, 13, 12 and 14 for Controls, Acute, Subacute and Chronic, respectively. RC nodes are shown in Table S2.3.

Control	Acute	Subacute	Chronic
rh-cuneus	rh-inferiorparietal	rh-inferiorparietal	rh-inferiorparietal
rh-inferiorparietal	rh-insula	rh-insula	rh-insula
rh-inferiortemporal	rh-posteriorcingulate	rh-lateralorbitofrontal	rh-lateraloccipital
rh-insula	rh-precentral	rh-paracentral	rh-posteriorcingulate
rh-lateraloccipital	rh-precuneus	rh-postcentral	rh-precentral
rh-lateralorbitofrontal	rh-superiorfrontal	rh-posteriorcingulate	rh-rostralmiddlefrontal
rh-paracentral	rh-superiorparietal	rh-precentral	rh-superiorfrontal
rh-posteriorcingulate	rh-superiortemporal	rh-precuneus	rh-superiorparietal
rh-precentral	lh-inferiorparietal	rh-rostralmiddlefrontal	lh-insula
rh-precuneus	lh-insula	rh-superiorfrontal	lh-posteriorcingulate
rh-rostralmiddlefrontal	lh-posteriorcingulate	rh-superiorparietal	lh-precentral
rh-superiorfrontal	lh-precentral	lh-insula	lh-precuneus
rh-superiorparietal	lh-precuneus	lh-lateraloccipital	lh-superiorfrontal
rh-supramarginal	lh-superiorfrontal	lh-paracentral	lh-superiorparietal
lh-inferiorparietal	lh-superiorparietal	lh-postcentral	
lh-insula		lh-posteriorcingulate	
lh-isthmuscingulate		lh-precentral	
lh-lingual		lh-precuneus	
lh-paracentral		lh-superiorfrontal	
lh-postcentral		lh-superiorparietal	
lh-posteriorcingulate			
lh-precentral			
lh-precuneus			
lh-rostralmiddlefrontal			
lh-superiorfrontal			
lh-superiorparietal			
lh-supramarginal			

Table S2.3 - Rich-club nodes for each group computed from binary DTI networks

An alternative method to statistically assess the rich-club subnetwork has been proposed which aims to exploit the maximum deviation of the normalised rich-club coefficient and assigns a unique *p*-value to assess the significance of the identified rich-club topology[14]. This method was implemented for binary networks, and has yet to be employed on a brain connectome. Its application on each of our cohort binary networks revealed the rich-club subnetwork to be non-significant ($p > 0.05$, Table S2.4).

	Control	Acute	Subacute	Chronic
p-value	0.678	0.781	0.314	0.758
deg_peak (degree at peak rich-club coefficient)	11	13	12	14
Peak (peak rich-club coefficient value)	0.0413	0.0309	0.1035	0.1071
Mean peak_rand (Average peak rich-club coeffient of 1000 random networks)	0.0828	0.1525	0.1037	0.2427

Table S2.4 - *p*-value of significant rich-club topology and rich-club coefficient parameters from an alternative rich-club statistical validation method[14].

There are several potential explanations for this null result which are inherent to networks constructed from brain imaging data. A primary reason is in employing binary networks (see full discussion in the

following section S2.3). However, the null results in themselves are important to strengthen the argument for weighted network approaches, which can provide additional biological information, in showing group differences.

2.3 Weighted versus binary networks discussion

A binary network is a fundamental representation of the brain, simply stating whether streamlines were tracked between pairwise regions. Such networks can introduce an arbitrary threshold when deciding how many streamlines must be present to be considered a connection. In comparison, weighted networks can capture the heterogeneity in brain connectivity, with weights corresponding to the number of streamlines from tractography, or the average values of diffusivity measures in the associated connections (e.g. fractional anisotropy or mean diffusivity). However, weighted networks may include spurious connections (without thresholding), but normalising the weights by the total number of tracts in an individual's connectome can help to "down-weight" connections with fewer tracts. Having such weights are closer to capturing the complexity of the brain with white matter pathways of varying tract size connecting different regions, leading to a broad range of possible connection strengths. For these reasons, diffusion MRI networks are typically weighted for analysis with this being the overall preference in diffusion network reliability studies[15–19], particularly as binary networks have demonstrated greater variability in network measures (such as clustering coefficient, characteristic path length, density/strength) when compared to weighted networks in reproducibility studies[17,18].

The biophysical properties captured in MRI data are informative of structural organisation and thus the brain benefits from the rich representation afforded by dense networks with a range of weights when compared to other kinds of networks (such as those based on internet, air travel or social networks). For example, given the high density of a brain network, topological features such as small-worldness are preserved in a weighted network, but are not observed in binary versions of similar density[18]. The omission of weight information will similarly affect a rich club analysis given that a rich club subnetwork is characterised by edges with high tract volume possessing high metabolic demand, and long spanning tracts with great inter-regional functional coupling[20–22]. These underlying biophysical features are indicative of a white matter pathway effective for information passing (and therefore connecting regions most important for network integration), and are embedded in the diffusion-related weights. We can observe this in our rich club analysis on weighted and binary DTI networks with equal densities. The binary networks had a greater degree range in which the rich club topology exists starting at a much lower degree and with lower k_{max}^{group} (compared to weighted networks), leading to more regions labelled as rich club nodes, with greater variance in these regions

across groups. Our results also highlight the need for alternative methods to introduce further rigour to rich-club analysis in brain network and until such methods are systematically validated on connectomes, our present supplementary analysis demonstrates the practicality in using *a priori* regions.

	Age at MRI			
	**Controls**	**mTBI Patients**		
Subject		Acute	Subacute	Chronic
1	12.82	13.23	Failed QC	14.36
2	14.90	20.29	20.33	21.41
3	12.00	12.91	12.95	14.16
4	13.35	17.47	17.51	18.56
5	15.84	13.11	13.16	14.25
6	14.21	13.64	No MRI	No MRI
7	12.62	11.51	Failed QC	12.65
8	13.61	13.44	13.48	14.56
9	Failed QC	No MRI	12.42	No MRI

Table S3 - Age (in years) at time of MRI for each subject in control and mTBI group analyzed. 'Failed QC' is when MRI data was removed from analyses due to excessive subject movement during acquisition.

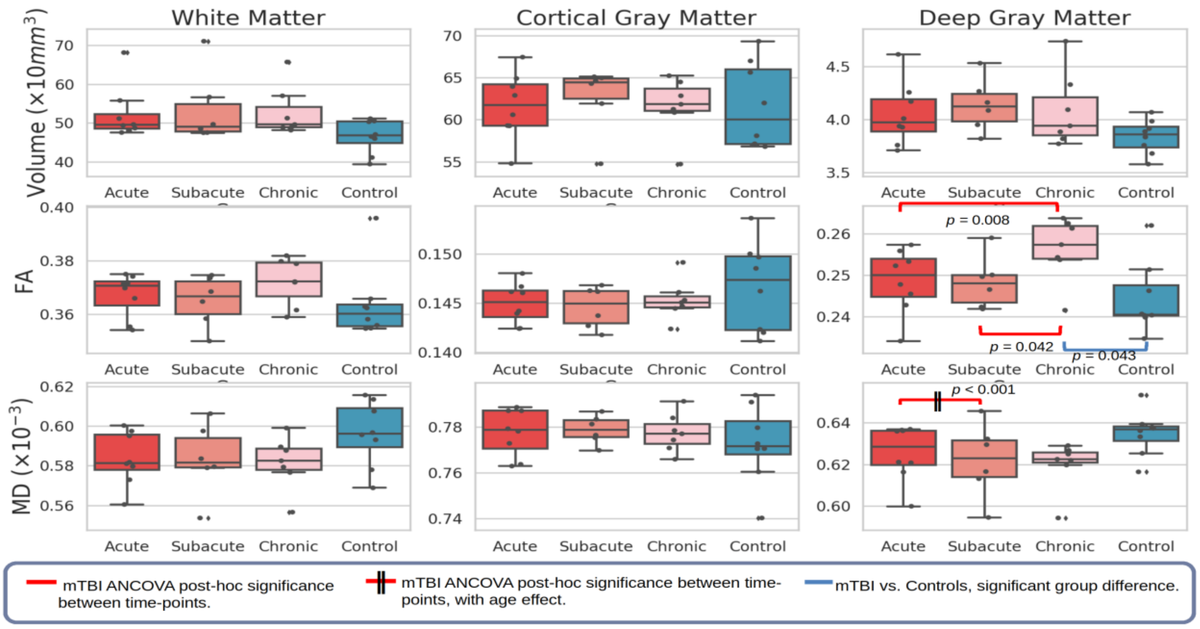

Figure S3 - Box plots of diffusion measures and T1-weighted brain volume with mTBI time-point and Controls in each whole brain segmentation masks. Individual data points are plotted as black circles. Significant post-hoc differences between time-points are shown only when the corresponding ANCOVA analysis in the mTBI cohort is significant.

		Longitudinal repeated measures ANCOVA													Cross-sectional ANCOVA											
												Post-hoc *p*-value between time points			Acute mTBI vs. Control						Chronic mTBI vs. Control					
		Age-adjusted mean						Fixed effects							Age-adjusted mean		Fixed effect significance				Age-adjusted mean		Fixed effect significance			
		Acute		Subacute		Chronic		Age at MRI		Time-point		Acute vs Subacute	Acute vs Chronic	Subacute vs Chronic	Controls		Age at MRI		Group		Controls		Age at MRI		Group	
		Mean	Std Error	Mean	Std Error	Mean	Std Error	F-statistic	*p*-value	F-statistic	*p*-value				Mean	Std Error	F-statistic	p-value	F-statistic	p-value	Mean	Std Error	F-statistic	p-value	F-statistic	p-value
T1-Weighted Volume (mm^3)	White matter	515694	28508	513468	32657	518101	25387	313.26	**<0.0005**	0.168	0.848				471750	15370	12.149	**0.004**	3.997	0.067	478540	16500	7.355	**0.019**	1.708	0.216
	Cortical GM	618910	14295	621082	12213	615949	11696	0.353	0.573	2.365	0.194				616100	16926	0.002	0.965	0	0.986	614525	17011	0.102	0.755	0.017	0.898
	Deep GM	40469	948	40997	758	40991	1039	0.012	0.92	0.935	0.435				38598	750	4.859	**0.046**	2.425	0.143	38884	903	3.251	0.097	0.963	0.346
FA	White matter	0.366	0.003	0.365	0.004	0.372	0.003	0.708	0.432	6.524	0.074				0.364	0.004	0.242	0.631	0.234	0.636	0.365	0.004	0.524	0.483	0.838	0.378
	Cortical GM	0.145	0.001	0.144	0.001	0.145	0.001	0.248	0.638	3.33	0.12				0.147	0.001	1.048	0.325	0.59	0.456	0.147	0.001	0.033	0.859	0.324	0.579
	Deep GM	0.249	0.003	0.248	0.003	0.257	0.003	0.016	0.905	8.806	**0.013**		0.008	0.042	0.245	0.003	1.496	0.243	0.634	0.44	0.245	0.003	0.232	0.639	5.111	**0.043**
MD x10^{-3}	White matter	0.584	0.004	0.583	0.003	0.581	0.004	13.633	**0.009**	2.269	0.199				0.594	0.004	8.227	**0.013**	2.207	0.161	0.592	0.005	7.894	**0.016**	0.761	0.4
	Cortical GM	0.777	0.003	0.777	0.002	0.777	0.003	0.016	0.903	0.012	0.988				0.773	0.005	0.451	0.514	0.33	0.575	0.772	0.005	0.008	0.928	0.426	0.526
	Deep GM	0.627	0.003	0.622	0.003	0.622	0.004	15.199	**0.009**	44.283	**0.001**	<0.001			0.633	0.003	1.196	**0.005**	1.944	0.187	0.632	0.004	6.141	**0.029**	2.894	0.115

Table S4 - Whole brain segment measures of diffusion properties and volume analyses. Age-adjusted means and statistical results from longitudinal (mTBI only) and cross-sectional (mTBI vs control) ANCOVA analyses. 'Age at MRI' is at the acute time-point for mTBI subjects. ANCOVA significance is at <= 0.05, marked in **bold.** GM = Gray matter.

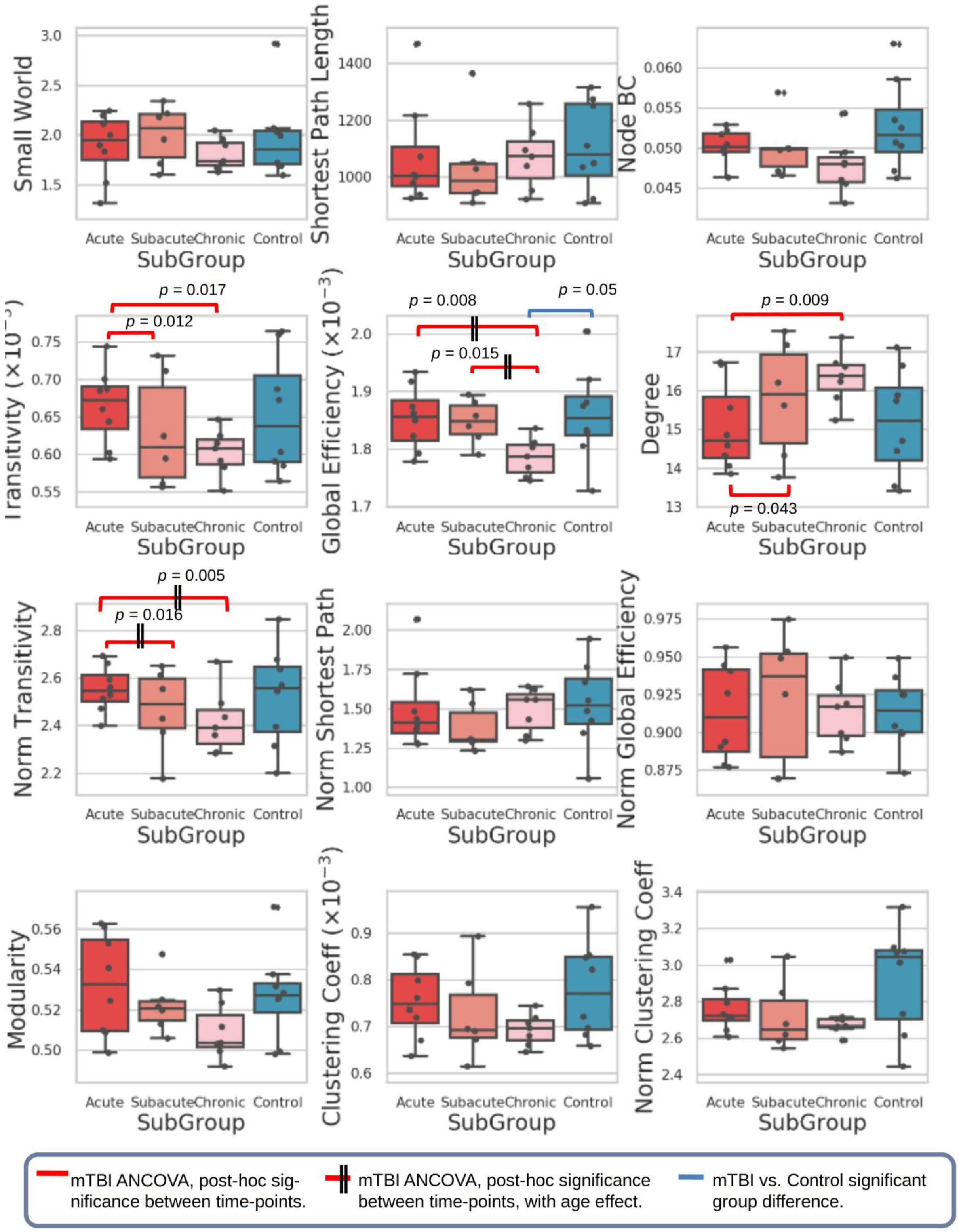

Figure S4 - Box plots of global network theoretical measures at each mTBI time-point and for Controls. Individual data points are plotted as black circles. Significant post-hoc differences between time-points are shown only when the corresponding ANCOVA analysis in the mTBI cohort is significant. BC = Betweenness centrality.

Longitudinal repeated measures ANCOVA

		Age-adjusted mean						Fixed effects				Post-hoc *p*-value between time points		
		Acute		Subacute		Chronic		Age at MRI		Time-point		Acute vs Subacute	Acute vs Chronic	Subacute vs Chronic
		Mean	Std Error	Mean	Std Error	Mean	Std Error	F-statistic	*p*-value	F-statistic	*p*-value			
	**Small world**	1.879	0.116	1.985	0.126	1.793	0.063	0.227	0.662	1.414	0.323			
	**Shortest path**	1075.96	66.103	1044.44	70.477	1075.475	44.320	0.010	0.923	0.091	0.914			
	**Transitivity (x10^{-3})**	0.674	0.019	0.619	0.025	0.603	0.013	0.038	0.860	21.884	**0.018**	0.012	0.017	
	**Global efficiency (x10^{-3})**	1.855	0.019	1.861	0.029	1.786	0.009	248.134	**0.003**	14.764	**0.004**		0.008	0.015
	**Node BC**	224.176	4.636	218.860	4.739	217.788	9.710	72.951	**0.004**	1.068	0.398			
	**Degree**	14.831	0.470	15.914	0.575	16.311	0.247	2.082	0.230	11.232	**0.023**	0.043	0.009	
	**Modularity**	0.532	0.009	0.520	0.005	0.502	0.008	2.573	0.338	3.449	0.119			
	**CC (x10^{-3})**	0.762	0.025	0.734	0.039	0.699	0.017	3.234	< **0.0005**	1.975	0.213			
Normalised	**Transitivity**	2.570	0.037	2.425	0.079	2.424	0.045	19.248	**0.018**	40.395	**< 0.0005**	0.016	0.005	
	**Shortest path**	1.504	0.093	1.383	0.067	1.498	0.053	0.276	0.625	0.842	0.473			
	**Global efficiency**	0.909	0.010	0.925	0.012	0.913	0.007	58.192	**0.002**	4.202	*0.065*	0.027		
	**CC**	2.781	0.050	2.757	0.085	2.669	0.017	0.558	0.489	2.290	0.163			

Cross-sectional ANCOVA

		Acute mTBI vs. Control						Chronic mTBI vs. Control					
		Age-adjusted mean		Fixed effect significance				Age-adjusted mean		Fixed effect significance			
		Controls		Age at MRI		Group		Controls		Age at MRI		Group	
		Mean	Std Error	F-statistic	p-value	F-statistic	p-value	Mean	Std Error	F-statistic	p-value	F-statistic	p-value
	**Small world**	1.972	0.139	0.248	0.627	0.225	0.643	1.966	0.123	0.026	0.874	0.828	0.381
	**Shortest path**	1101.20	62.04	0.545	0.474	0.052	0.824	1106.358	51.965	0.013	0.912	0.221	0.647
	**Transitivity (x10^{-3})**	0.647	0.022	3.693	0.077	0.513	0.487	0.650	0.022	0.869	0.370	1.810	0.203
	**Global efficiency (x10^{-3})**	1.860	0.026	0.043	0.839	0.038	0.848	1.861	0.024	0.186	0.674	4.760	**0.050**
	**Node BC**	232.745	6.945	0.086	0.774	1.060	0.322	233.736	7.753	0.442	0.519	4.152	*0.064*
	**Degree**	15.251	0.444	1.332	0.269	0.140	0.714	15.190	0.414	0.013	0.911	3.478	0.087
	**Modularity**	0.526	0.009	1.119	0.309	0.387	0.545	0.527	0.007	0.002	0.967	2.948	0.112
	**CC (x10^{-3})**	0.771	0.030	4.377	*0.057*	0.054	0.821	0.775	0.029	1.133	0.308	3.308	0.094
Normalised	**Transitivity**	2.519	0.600	0.143	0.711	0.168	0.688	2.522	0.067	0.000	0.990	1.155	0.304
	**Shortest path**	1.516	0.096	1.125	0.308	0.000	0.998	1.524	0.082	0.191	0.670	0.056	0.817
	**Global efficiency**	0.916	0.010	1.818	0.201	0.084	0.776	0.915	0.008	1.211	0.293	0.050	0.827
	**CC**	2.908	0.081	1.104	0.312	1.382	0.261	2.913	0.080	0.249	0.627	4.091	*0.066*

Table S5 - Global network theory analyses. Age-adjusted means and statistical results from longitudinal (mTBI only) and cross-sectional (mTBI vs control) ANCOVA analyses. ANCOVA significance is at $p <= 0.05$, marked in **bold,** trends are *italicised*. 'Age at MRI' is at the acute time point for mTBI subjects. Normalised network measures are calculated against 1000 random networks of equal density to the observed networks. BC = Betweenness Centrality; CC = Clustering Coefficient.

		Longitudinal repeated measures ANCOVA													Cross-sectional ANCOVA											
												Post-hoc *p*-value between time			Acute mTBI vs. Control						Chronic mTBI vs. Control					
		Age-adjusted mean						Fixed effects				Acute vs Subacute	Acute vs Chronic	Subacute vs Chronic	Age-adjusted mean		Fixed effect significance				Age-adjusted mean		Fixed effect significance			
		Acute		Subacute		Chronic		Age at MRI		Time-point					Controls		Age at MRI		Group		Controls		Age at MRI		Group	
		Mean	Std Error	Mean	Std Error	Mean	Std Error	F-statistic	*p*-value	F-statistic	*p*-value				Mean	Std Error	F-statistic	p-value	F-statistic	p-value	Mean	Std Error	F-statistic	p-value	F-statistic	p-value
**NoE**	Rich club	27.50	0.73	23.92	2.16	29.16	0.98	24.96	0.287	2.733	0.210				27.52	0.84	13.08	**0.003**	0.207	0.657	27.28	1.04	13.08	0.316	1.197	0.295
	Feeder	188.30	3.86	198.01	5.65	198.20	5.29	0.700	0.444	8.250	**0.047**	0.074	0.066		184.54	5.25	1.76	0.208	0.370	0.553	183.96	5.96	1.758	0.693	2.443	0.144
	Local	291.48	10.71	313.98	15.20	327.19	5.80	4.094	0.143	5.315	**0.050**		0.020		306.47	10.71	0.48	0.500	0.624	0.444	305.50	10.01	0.482	1.000	1.442	0.251
**NoS**	Rich club	98.50	5.18	115.15	6.46	103.87	7.50	4.087	0.085	8.283	**0.029**	0.017			86.78	4.88	5.64	**0.034**	2.443	0.142	86.64	6.08	2.602	0.133	3.865	0.073
	Feeder	42.42	1.92	37.37	2.09	40.65	2.17	0.672	0.442	6.306	0.100				40.01	1.78	1.08	0.317	0.268	0.614	40.28	1.85	3.961	0.070	0.236	0.636
	Local	36.70	2.05	32.28	1.46	34.23	1.91	5.768	0.075	4.942	*0.063*				32.29	1.73	2.20	0.162	1.598	0.228	32.20	1.51	1.685	0.217	1.108	0.312
**FA**	Rich club	0.467	0.003	0.467	0.006	0.468	0.005	0.149	0.715	0.051	0.951				0.458	0.004	0.146	0.709	3.454	0.086	0.457	0.004	1.853	0.198	4.353	*0.059*
	Feeder	0.422	0.004	0.426	0.004	0.428	0.003	1.291	0.304	4.116	0.094				0.419	0.004	0.265	0.616	0.871	0.368	0.418	0.004	1.121	0.310	2.909	0.114
	Local	0.397	0.005	0.403	0.005	0.409	0.004	0.555	0.485	9.658	**0.017**		0.005		0.394	0.005	0.010	0.920	0.356	0.561	0.394	0.005	0.475	0.503	4.077	*0.065*
**MD x10^{-3}**	Rich club	0.560	0.003	0.559	0.004	0.562	0.004	37.871	**0.001**	1.946	0.220				0.572	0.003	6.963	**0.020**	4.914	**0.045**	0.572	0.004	1.849	0.199	3.756	0.076
	Feeder	0.580	0.006	0.583	0.008	0.579	0.006	97.821	**0.003**	1.289	0.328				0.588	0.004	4.385	*0.056*	2.356	0.149	0.588	0.004	3.919	0.071	2.098	0.173
	Local	0.588	0.006	0.583	0.004	0.586	0.006	2985.06	**<0.0005**	4.990	**0.044**	0.016			0.598	0.005	5.454	**0.036**	1.704	0.214	0.599	0.005	4.110	0.064	2.734	0.122
**AD x10^{-3}**	Rich club	0.874	0.005	0.870	0.007	0.877	0.007	6.034	**0.051**	0.752	0.513				0.883	0.005	8.165	**0.013**	0.950	0.348	0.883	0.006	4.700	*0.051*	0.834	0.379
	Feeder	0.861	0.004	0.865	0.006	0.865	0.005	13.167	**0.010**	0.614	0.573				0.873	0.004	12.886	**0.003**	2.404	0.145	0.873	0.005	11.034	**0.006**	0.635	0.441
	Local	0.855	0.003	0.856	0.005	0.862	0.004	16.007	**0.010**	2.957	0.141				0.867	0.004	16.174	**0.001**	2.813	0.117	0.867	0.005	12.986	**0.003**	0.570	0.464
**RD x10^{-3}**	Rich club	0.404	0.003	0.401	0.005	0.405	0.004	3.407	0.114	0.958	0.443				0.416	0.003	2.516	0.137	6.241	**0.027**	0.416	0.004	0.174	0.684	5.392	**0.039**
	Feeder	0.437	0.004	0.436	0.005	0.434	0.003	0.361	0.580	0.881	0.466				0.445	0.005	1.413	0.256	1.844	0.198	0.445	0.005	1.034	0.329	2.626	0.131
	Local	0.456	0.006	0.448	0.004	0.449	0.006	11.489	**0.022**	7.424	**0.023**	0.024	0.028		0.464	0.006	2.207	0.161	1.129	0.307	0.464	0.006	1.279	0.279	3.506	0.084

Table S6 - Rich-club and subnetwork analyses. Age-adjusted means and statistical results from longitudinal (mTBI only) and cross-sectional (mTBI vs control) ANCOVA analyses. ANCOVA significance is at *p* <= 0.05, marked in **bold,** trends are *italicised*. 'Age at MRI' is at the acute time point for mTBI subjects. NoE = number of edges in the subnetwork; NoS = Number of streamlines.

1. Wang, R., Benner, T., Sorensen, A. & Wedeen, V. Diffusion Toolkit: A Software Package for Diffusion Imaging Data Processing and Tractography. in *International Society for Magnetic Resonance in Medicine* 3720 (2007).
2. Hess, C. P., Mukherjee, P., Han, E. T., Xu, D. & Vigneron, D. B. Q-ball reconstruction of multimodal fiber orientations using the spherical harmonic basis. *Magn. Reson. Med. Off. J. Soc. Magn. Reson. Med. Soc. Magn. Reson. Med.* **56**, 104–117 (2006).
3. Schirmer, M. D. & Chung, A. W. Structural subnetwork evolution across the life-span: rich-club, feeder, seeder. in *Connectomics in Neuroimaging Workshop* 134–143 (LNCS, Springer, 2018). doi:https://doi.org/10.1007/978-3-030-00755-3_15.
4. Schirmer, M. D., Chung, A. W., Grant, P. E. & Rost, N. S. Network structural dependency in the human connectome across the life-span. *Netw. Neurosci.* 1–30 (2019) doi:10.1162/netn_a_00081.
5. van den Heuvel, M. P. *et al.* Abnormal rich club organization and functional brain dynamics in schizophrenia. *JAMA Psychiatry* **70**, 783–792 (2013).
6. Heuvel, M. P. van den & Sporns, O. Rich-Club Organization of the Human Connectome. *J. Neurosci.* **31**, 15775–15786 (2011).
7. Grayson, D. S. *et al.* Structural and functional rich club organization of the brain in children and adults. *PloS One* **9**, e88297 (2014).
8. Verhelst Helena, Vander Linden Catharine, De Pauw Toon, Vingerhoets Guy & Caeyenberghs Karen. Impaired rich club and increased local connectivity in children with traumatic brain injury: Local support for the rich? *Hum. Brain Mapp.* **0**, (2018).
9. Rubinov, M. & Sporns, O. Complex network measures of brain connectivity: Uses and interpretations. *NeuroImage* **52**, 1059–1069 (2010).
10. Opsahl, T., Colizza, V., Panzarasa, P. & Ramasco, J. J. Prominence and control: the weighted rich-club effect. *Phys. Rev. Lett.* **101**, 168702 (2008).
11. Rubinov, M. & Sporns, O. Weight-conserving characterization of complex functional brain networks. *NeuroImage* **56**, 2068–2079 (2011).

12. Colizza, V., Flammini, A., Serrano, M. A. & Vespignani, A. Detecting rich-club ordering in complex networks. *Nat. Phys.* **2**, 110–115 (2006).

13. Maslov, S. & Sneppen, K. Specificity and stability in topology of protein networks. *Science* **296**, 910–913 (2002).

14. Muscoloni, A. & Cannistraci, C. V. Rich-clubness test: how to determine whether a complex network has or doesn't have a rich-club? *ArXiv170403526 Cond-Mat Physicsphysics* (2017).

15. Zalesky, A., Fornito, A. & Bullmore, E. T. Network-based statistic: identifying differences in brain networks. *NeuroImage* **53**, 1197–1207 (2010).

16. Welton, T., Kent, D. A., Auer, D. P. & Dineen, R. A. Reproducibility of Graph-Theoretic Brain Network Metrics: A Systematic Review. *Brain Connect.* **5**, 193–202 (2015).

17. Yuan, J. P. *et al.* Test–Retest Reliability of Graph Theoretic Metrics in Adolescent Brains. *Brain Connect.* (2018) doi:10.1089/brain.2018.0580.

18. Colon-Perez, L. M., Couret, M., Triplett, W., Price, C. C. & Mareci, T. H. Small Worldness in Dense and Weighted Connectomes. *Front. Phys.* **4**, (2016).

19. Messaritaki, E., Dimitriadis, S. I. & Jones, D. K. Optimization of graph construction can significantly increase the power of structural brain network studies. *NeuroImage* **199**, 495–511 (2019).

20. Heuvel, M. P. van den, Kahn, R. S., Goñi, J. & Sporns, O. High-cost, high-capacity backbone for global brain communication. *Proc. Natl. Acad. Sci.* **109**, 11372–11377 (2012).

21. Collin, G., Sporns, O., Mandl, R. C. W. & van den Heuvel, M. P. Structural and functional aspects relating to cost and benefit of rich club organization in the human cerebral cortex. *Cereb. Cortex N. Y. N 1991* **24**, 2258–2267 (2014).

22. Griffa, A. & Van den Heuvel, M. P. Rich-club neurocircuitry: function, evolution, and vulnerability. *Dialogues Clin. Neurosci.* **20**, 121–132 (2018).

2. Hess, C. P., Mukherjee, P., Han, E. T., Xu, D. & Vigneron, D. B. Q-ball reconstruction of multimodal fiber orientations using the spherical harmonic basis. *Magn. Reson. Med. Off. J. Soc. Magn. Reson. Med. Soc. Magn. Reson. Med.***56**, 104–117 (2006).
3. Schirmer, M. D. & Chung, A. W. Structural subnetwork evolution across the life-span: rich-club, feeder, seeder. in *Connectomics in Neuroimaging Workshop* 134–143 (LNCS, Springer, 2018). doi:https://doi.org/10.1007/978-3-030-00755-3_15
4. Schirmer, M. D., Chung, A. W., Grant, P. E. & Rost, N. S. Network structural dependency in the human connectome across the life-span. *Netw. Neurosci.* 1–30 (2019). doi:10.1162/netn_a_00081
5. van den Heuvel, M. P. *et al.* Abnormal rich club organization and functional brain dynamics in schizophrenia. *JAMA Psychiatry***70**, 783–792 (2013).
6. Heuvel, M. P. van den & Sporns, O. Rich-Club Organization of the Human Connectome. *J. Neurosci.***31**, 15775–15786 (2011).
7. Grayson, D. S. *et al.* Structural and functional rich club organization of the brain in children and adults. *PloS One***9**, e88297 (2014).
8. Verhelst Helena, Vander Linden Catharine, De Pauw Toon, Vingerhoets Guy & Caeyenberghs Karen. Impaired rich club and increased local connectivity in children with traumatic brain injury: Local support for the rich? *Hum. Brain Mapp.***0**, (2018).
9. Rubinov, M. & Sporns, O. Complex network measures of brain connectivity: Uses and interpretations. *NeuroImage***52**, 1059–1069 (2010).
10. Opsahl, T., Colizza, V., Panzarasa, P. & Ramasco, J. J. Prominence and control: the weighted rich-club effect. *Phys. Rev. Lett.***101**, 168702 (2008).
11. Rubinov, M. & Sporns, O. Weight-conserving characterization of complex functional brain networks. *NeuroImage***56**, 2068–2079 (2011).
12. Colizza, V., Flammini, A., Serrano, M. A. & Vespignani, A. Detecting rich-club ordering in complex networks. *Nat. Phys.***2**, 110–115 (2006).

13. Maslov, S. & Sneppen, K. Specificity and stability in topology of protein networks. *Science***296**, 910–913 (2002).

14. Zalesky, A., Fornito, A. & Bullmore, E. T. Network-based statistic: identifying differences in brain networks. *NeuroImage***53**, 1197–1207 (2010).

15. Welton, T., Kent, D. A., Auer, D. P. & Dineen, R. A. Reproducibility of Graph-Theoretic Brain Network Metrics: A Systematic Review. *Brain Connect.***5**, 193–202 (2015).

16. Yuan, J. P. *et al.* Test–Retest Reliability of Graph Theoretic Metrics in Adolescent Brains. *Brain Connect.* (2018). doi:10.1089/brain.2018.0580

17. Colon-Perez, L. M., Couret, M., Triplett, W., Price, C. C. & Mareci, T. H. Small Worldness in Dense and Weighted Connectomes. *Front. Phys.***4**, (2016).

18. Messaritaki, E., Dimitriadis, S. I. & Jones, D. K. Optimization of graph construction can significantly increase the power of structural brain network studies. *NeuroImage***199**, 495–511 (2019).

19. Heuvel, M. P. van den, Kahn, R. S., Goñi, J. & Sporns, O. High-cost, high-capacity backbone for global brain communication. *Proc. Natl. Acad. Sci.***109**, 11372–11377 (2012).

20. Collin, G., Sporns, O., Mandl, R. C. W. & van den Heuvel, M. P. Structural and functional aspects relating to cost and benefit of rich club organization in the human cerebral cortex. *Cereb. Cortex N. Y. N 1991***24**, 2258–2267 (2014).

21. Griffa, A. & Van den Heuvel, M. P. Rich-club neurocircuitry: function, evolution, and vulnerability. *Dialogues Clin. Neurosci.***20**, 121–132 (2018).

22. Muscoloni, A. & Cannistraci, C. V. Rich-clubness test: how to determine whether a complex network has or doesn't have a rich-club? *ArXiv170403526 Cond-Mat Physicsphysics* (2017).
